# Cholinergic Mechanisms of Target Oddball Stimuli Detection: The Late “P300-Like” Event-Related Potential in Rats

**DOI:** 10.1155/2018/4270263

**Published:** 2018-10-16

**Authors:** A. Ahnaou, R. Biermans, W. H. I. M. Drinkenburg

**Affiliations:** Department of Neuroscience, Janssen Research & Development, Janssen Pharmaceutica NV, Turnhoutseweg 30, 2340 Beerse, Belgium

## Abstract

Event-related potentials (ERPs) and oscillations (EROs) provide powerful tools for studying the brain's synaptic function underlying information processing. The P300 component of ERPs indexing attention and working memory shows abnormal amplitude and latency in neurological and psychiatric diseases that are sensitive to pharmacological agents. In the active auditory oddball discriminant paradigm, behavior and auditory-evoked potentials (AEPs) were simultaneously recorded in awake rats to investigate whether P300-like potentials generated in rats responding to rare target oddball tones are sensitive to subcutaneous modulation of the cholinergic tone by donepezil (1 mg/kg) and scopolamine (0.64 mg/kg). After operant training, rats consistently discriminate rare target auditory stimuli from frequent irrelevant nontarget auditory stimuli by a higher level of correct lever presses (i.e., accuracy) in target trials associated with a food reward. Donepezil attenuated the disruptive effect of scopolamine on the level of accuracy and premature responses in target trials. Larger P300-like peaks with early and late components were revealed in correct rare target stimuli trials as compared to frequent tones. Donepezil enhanced the peak amplitude of the P300-like component to target stimuli and evoked slow theta and gamma oscillations, whereas scopolamine altered the amplitude of the P300-like component and EROs to target stimuli. Pretreatment with donepezil attenuated effects of scopolamine on the peak amplitude of the P300-like component and on EROs. This study provides evidence that AEP P300-like responses can be elicited by rats engaged in attentive and memory processing of target stimuli and outline the relevance of the cholinergic system in stimulus discrimination processing. The findings highlight the sensitivity of this translational index for investigating brain circuits and/or novel pharmacological agents, which modulate cholinergic transmission associated with increased allocation of attentional resources.

## 1. Introduction

Despite the recent flurry of research efforts in contemporary cognitive neuroscience, there remains a need to identify an early biomarker with high sensitivity and specificity for cognitive impairments. Many cognitive functions are unique to humans and cannot be addressed in experimental animals whilst cognitive paradigms and models must have translational validity to be of use in drug discovery. Animal models are often discussed regarding face, constructive, and predictive validity criteria, and the better an animal model fulfils these criteria, the more successful the translation of preclinical data to clinical applications is likely to be. Despite the availability of some level of face and construct validity for assays and disease models used in cognition research, the critical feature for drug discovery is the predictive validity [1–3]. Ideal animal models of cognition should provide a prediction of the specific conditions for which compounds would be most useful and could be combined with toxicology and safety pharmacology models to estimate the therapeutic effect [4]. A recent promising project seeking to develop new ways to classify mental disorders proposes a new research domain criteria (RDoC) approach that may facilitate translational research. Focus on brain neuronal circuits and connectivity is a promising approach to provide a closer link between animal models and the disorders they model [5–9].

Cognition refers to mental processes that are involved in gaining knowledge and comprehension such as problem-solving and decision-making, attention, memory and working memory, judgment, evaluation, and production of language. Neurons do not generally act in isolation but act as synchronized and rhythmically active assemblies to encode, transmit, and modulate information underpinning cognitive function under normal or evoked modalities. Thus, cognition can better be understood in terms of complex disparate networks operating over multiple temporal scales and incorporating diverse dimensions. Changes in the pattern of synaptic weights underlie the efficacy of communication neuronal ensembles and the process of long-term potentiation (LTP), an electrophysiological basic correlate of learning and memory [10–13]. Although mostly studied in hippocampal and neocortex animal preparations [14–16], rapid visual or auditory tetanus stimuli elicited changes in amplitude of event-related potentials (ERPs) recorded from the scalp of adult humans that are suggestive of LTP-like characteristics that are seen in experimental animals [17–19]. ERP components are voltage changes in the electroencephalographic activity that are specifically linked to a physical or mental stimulus, which provide a direct window into the brain mechanisms that are reflected in performance measures during attention processing paradigms in normal and disease conditions [20–23]. The temporal encoding of information is essential for synaptic plasticity, and the temporal immediacy of ERPs is advantageous in the study of memory encoding and retrieval processes.

In the oddball discrimination-task paradigm, the evoked ERP waveforms defined by their polarity and latency reflect a cascade of information processing of a stimuli that are functionally specified to attentional and memory mechanisms. The early P1-N1-P2 complex component reflects a preattentional response following the initial memory encoding of the stimulus characteristics [24, 25], whereas the N2 and P300 components are only elicited following selective attentional allocation to target stimuli [26]. The P300 or (P3) peak is a scalp positivity bioelectric ERP correlate that is elicited by low-probability task-relevant stimuli during stimulus classification tasks in auditory, visual, and somatosensory modalities. It occurs at approximately 300 ms after stimuli onset in humans. In rodents, there is a reduced latency of this signal due to the shorter distance that the signal must travel in the smaller brain of rodents. It can be classed as an “endogenous” component in the sense that it relies on the processing of the stimulus context and the levels of attention and arousal [23, 26, 27]. Following an initial sensory processing, an attention-driven comparison process evaluates the representation of the previous event in the working memory. The P300 peak amplitude and latency are modulated by a variety of factors, including subjective probability, stimulus saliency, availability of attentional resources, and it appears to be generated by a network of neural regions; the inferotemporal, perirhinal, prefrontal, cingulate, superior temporal, and parietal cortices, as well as the hippocampus.

The ERP P300 component is regarded as a neurophysiological indicator of cognitive processing of a stimulus [27]. In neurological and psychiatric disorders, a key prerequisite would be a biomarker that can reliably predict a transition and progression of the disease and facilitate early pharmacological intervention. Several neurological and psychiatric studies demonstrate the links between memory and cognitive impairments and negative changes observed in P300 peak latency and amplitude. P300 has emerged as a viable endophenotype for schizophrenia and may provide a robust marker for illness progression in an individual patient considered at risk of developing schizophrenia [28–30]. P300 peak delayed latency can serve to produce biomarkers of mild cognitive impairments [31, 32], predictive measure of brain amyloid load in preclinical Alzheimer's disease [33], and dementia and its progression [34, 35].

The P300 component amplitude is thought to be related to the processing and brain activity involved in the working memory. The most common theory is the context updating theory, which reflects the information processing involved when incoming information is integrated with a memory representation of a stimulus and the context with which it occurs. The latency with which the P300 wave occurs is thought to be negatively correlated to mental function. The latency shows the processing time needed before a response can be generated; hence, the later appearance of the peak shows a direct link to slowing of cognitive function [26]. Patients that have a damage in the temporoparietal junction have the auditory P300 response eliminated, which suggests that this neocortical region is potentially critical in the generation or propagation of the scalp P300 [36].

In addition to ERP waveforms, event-related oscillations (EROs) synchrony quantifies the time-locked change in the power of the EEG spectral contents associated with the event-related trials. Oscillations can be evoked (phase-locked to the stimulus) or induced (nonphase-locked to the stimulus). Functionally, EROs are not yet fully understood, as complex and integrative brain functions seem to rely on the superimposition of several oscillations [36]. Neuronal oscillations play a fundamental role in linking and coordinating interlinked and overlapping cerebral regions. Oscillatory rhythms in the theta and gamma frequency ranges are related to episodic memory and are widely used to monitor oscillations driven by hippocampal regions in coordination with the frontal cortical areas. Additionally, various neuromodulators such as the neurotransmitter acetylcholine (ACh) play key roles in influencing cognitive performance. For instance, the prefrontal cortex (PFC) and hippocampus receive a rich cholinergic input and are also heavily innervated by serotonergic, dopaminergic, noradrenergic, and histaminergic neurons [37]. Cholinergic transmission is the main target for most of the current symptomatic therapies in neurodegenerative diseases, in the form of ACh esterase (AChE) inhibitors such as donepezil. The pharmacological model based on scopolamine, which temporarily blocks muscarinic cholinergic receptors in the brain, is widely regarded as a standard model for investigating cognitive deficits and may be useful in the search for improved procognitive drugs. Despite the peripheral side effects that occur at higher doses of scopolamine, the primary effects on stimulus discrimination and attention make this compound good for pharmacological validation of the two-tone discrimination task.

Over the last decade, animal models of the long-latency ERP P300 component have been developed in cats [38] and non-human primates [39]. In rodents, studies using the passive oddball, where animals do not require extensive training, revealed late positive potentials in the mouse [40] and rat cortex [41–43]. In the active oddball paradigm, late positive components with peak latency at approximately 200–500 ms were identified in electrodes overlying cortical, hippocampal, and nucleus accumbens- and amygdala-recording sites [43, 44]. This was clearly identified in rats that paid attention to target stimuli in the active oddball, whereas it was not revealed in animals not performing in the passive oddball paradigm [41, 45]. In addition, the rat's ERP P300-like response was sensitive to pharmacological modulation of the cholinergic tone [46], which is consistent with changes observed in healthy human studies because of diminished cholinergic neurotransmission by scopolamine or a selective muscarinic receptor antagonist [47, 48].

Donepezil has been shown to attenuate scopolamine-induced reduction of the P300 amplitude, which is consistent with the modulation of P300 by donepezil in AD Alzheimer's patients [49]. It is important to note that the translational ability of the rat P300-like response to human P300 responses depends not on the polarity of the component, but on the location of the source of the activity and the morphological properties of its surrounding tissue, which are key to determining the cortical polarity of an ERP component [50]. Habituation during tasks has not been found to be a problem, with the P300-like response not changing dramatically for the number of trials required to obtain stable EEG averages [51].

The present study used an active oddball paradigm to demonstrate that P300-like responses and related EROS could be generated in rats under baseline conditions and to confirm that this P300-like response of attentive and memory processing is sensitive to modulation of cholinergic neurotransmission.

## 2. Materials and Methods

### 2.1. Animal Husbandry

All experimental procedures were conducted in strict accordance with the guidelines of the Association for Assessment and Accreditation of Laboratory Animal Care International (AAALAC) and with the European Communities Council Directive of 24th November 1986 (86/609/EEC) and were approved by the local ethical committee. The experiments were carried out on male adult Long-Evans rats (Harlan, Netherlands), weighing 300 g at the time of surgery and were housed in full-view Plexiglas (25 × 33 × 18 cm) individually ventilated cages (IVCs). All rats were provided with a microchip for identification and maintained under controlled environmental conditions, temperature 22°C ± 2°C, and humidity 60%, in a reverse 12-hour light-dark cycle (lights off at 07 : 00, lights on at 19 : 00). All experiments were conducted during the dark phase of the circadian rhythm. Once animals were fully grown, they were placed on a food-restricted diet of 20 g on nontask days and 15 g on task days, with water ad libitum and food available ad libitum on weekends, removed 24 h before the next training or test session. Weights of the animals were regularly monitored.

### 2.2. Two-Tone Auditory Discrimination Task (TTADT) Training

**Figure 1:**
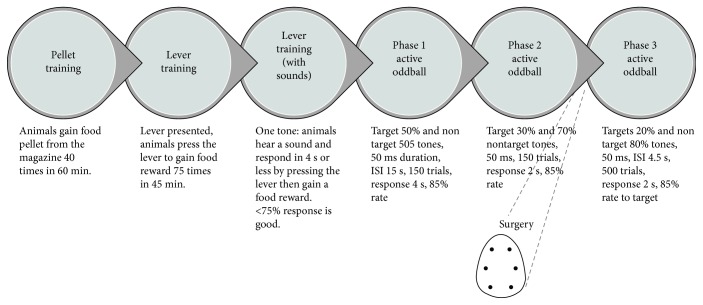
Schematic presentation of different phases of the active oddball discriminant task. Animals had to correctly respond to infrequent target (oddball) tones whilst correctly ignoring frequent standard tones. The criteria for good behavioral performance was >80% response to target and <20% response to standard tones. Epidural electrodes implanted in the rats recorded continuous EEG from specific brain areas (frontal, parietal, and occipital).

The training protocol is presented in Figure 1. In brief, animals first receive two to three food dispenser and magazine training sessions (operant box, Med Associates). The rats could explore the chamber, approach the food magazine, and eat the dustless precision pellets (BIOSERV, Frenchtown, NJ, USA). Magazine training sessions lasted for 60 min or until 40 pellets in the tray had been consumed, whichever occurred first (Figure 1). In the next session, rats were trained to press a lever for the food pellets reward under a fixed ratio-1 schedule of reinforcement. One tone (8 kHz target tone) was then presented, and animals were required to hear the tone and respond by lever pressing within 4 s to gain the food reward. A response of >75% within 4 s was considered and failure to press the lever within 4 s resulted in a timeout period of 15 s with house light off if they lever-pressed in the interstimulus interval (ISI) to discourage this behavior. If animals had a low number of lever presses in the ISI, then they progressed onto the active oddball phase 1. A total of 150 trials were presented to the animal in a 50/50 probability of occurrence for target tones (8 kHz) and nontarget tones (4 kHz) at 90 dB, 20 ms. Response to the target tone by pressing the lever within 4 s resulted in a food pellet reward, which would be a correct target trial. No reward was given for a lever press on the standard tone. Animals were required to have a response rate of ≥85% to the target tone with a standard response at ≤50%. Animals then progressed to phase 2, with the probability of a target tone reduced to 30%, and response to this target tone reduced to 2 s. Progression criteria were the same as for phase 1. Phase 3 had 500 trials, 20% probability of target tone, with 4.5 s ISI, and a response within 2 s of the target tone. After the training sessions, animals were chronically implanted with 6 epidural electrodes in the fronto-parieto-occipital areas.

### 2.3. Surgical Procedure

The surgery was performed as previously described [52]. Briefly, animals were equipped with 6 active electrodes over the right and left hemispheres (auditory cortex: ±2 mm posterior to Bregma and ±2 mm lateral to the midline; parietal cortex: −3 mm posterior to Bregma and ±4 mm lateral to the midline; and the occipital cortex −6.5 mm posterior to Bregma and ±4 mm lateral to the midline) and referenced to the ground electrode placed on the cerebellum. Electrodes were connected to a pin with a small insert and fitted into a 10-hole connector. The whole assembly was fixed with dental cement to the cranium.

### 2.4. Two-Tone Auditory Discrimination Task (TTADT) under Pharmacological Procedure

After a two-week recovery period, animals were placed in the operant box and connected to a wire to enable EEG recording. Animals were presented with one to two sessions of phase 3 training under the same conditions as before (500 trials, 20% probability of target tone, with 4.5 s ISI, and response within 2 s to target tones). Simultaneous EEG and behavioral response measurements started first with a silent recording run followed by a baseline recording run carried out during the task performance with nontarget and target tones. Continuous EEG were acquired at 2 kHz sample rate with an input range of ±500 mV through a Biosemi ActiveTwo system (Biosemi, Amsterdam, Netherlands) referenced to the CMS-DRL ground.

Afterwards, animals received combined pharmacological treatments with an interval of 30 min between both injections (group 1: vehicle + vehicle; group 2: donepezil + vehicle; group 3: vehicle + scopolamine; and group 4: donepezil + scopolamine). The postdrug run of 250 trials and behavioral recordings run started 30 min later.

Auditory stimuli were generated with a custom program written in LabVIEW, and sound stimuli were delivered through a speaker mounted above the floor of the experimental box. Sound intensity was calibrated with a sound meter 80 dB SPL in different locations of the recording box. In this active oddball paradigm, two stimulus conditions (target and nontarget) were pseudo-randomly presented with the restriction that consecutive target tones were separated by at least three nontarget tones. Continuous EEG were acquired at 2 kHz sample rate with an input range of ±500 mV through a Biosemi ActiveTwo system (Biosemi, Amsterdam, Netherlands) referenced to the CMS-DRL ground.

### 2.5. Data Analysis

#### 2.5.1. Behavioral Analysis

Scores were determined for correct target and correct nontarget trials, and numbers of premature responses (pressing lever during ITI) were used to calculate the percentage of responses. A full correct response was deemed as the rat responding to the target tone within 2 sec and gaining the food reward. Incomplete responses occurred if the animal had pressed the lever but failed to gain the food reward, and incorrect responses were considered.

#### 2.5.2. EEG Analysis

The peak amplitudes of the ERP waveforms and EROs were computed in artefact-free EG intervals, which were bandpass-filtered using a zero-phase lag filter with a 48 dB/octave roll-off. Stimulus-locked trials were extracted for all instances where there was a correct behavioral response for each of the two experimental conditions (nontarget and rare target stimuli), with each trial comprising up to 800 ms of data and − 100 ms prestimulus. The response window for correct responses on the target trials was 200–500 ms. A semi-automated algorithm was used to select discernable ERP peaks, which were baseline-corrected using the 100 ms prestimulus interval. Waveform averages were generated for correct target and correct nontarget trials for each animal, and grand average AEP waveforms for each of the components were computed across groups. The peak amplitudes and latencies of the P300-like components were calculated for each rat and condition. Additionally, event-related oscillations (EROs) were computed using the multitaper Morlet wavelet analysis, which offers superior time resolution to identify rapid changes in spectral components locked to stimuli during performance in the discriminant behavioral task. Trial-by-trial time-frequency decomposition was applied in the frequency range of 1–50 Hz (0.25 Hz steps). Wavelet transformation was applied to each single trial. The absolute power for each single trial wavelet cycle was then computed by taking the square of the magnitude of the complex valued for each trial, and these were then averaged across all trials to produce a resultant EROS that was termed event-induced oscillations per electrode site per subject.

### 2.6. Drugs

Donepezil (Ningbo Distant Chemicals Co., Ltd, China) and scopolamine (Enzo Life Sciences International Inc., USA) were dissolved in sterile 0.9% saline solution and were administered subcutaneously (sc) in a volume of 10 ml/kg body weight. Saline was administered in control groups. Donepezil (1.0 mg/kg) and scopolamine (0.16 mg/kg) were administered at the doses that showed efficacy in neurophysiological and behavioral studies in rodents [53, 54]. In reversal pharmacological conditions, donepezil was given 30 min prior to scopolamine.

### 2.7. Statistical Analysis

Behavioral and AEP P300 amplitude and latency data were analyzed independently using a repeated-measures one-way ANOVA test. Scores for correct target and nontarget trials, number of premature responses, waveform peaks, and latencies were presented as mean ± SEM, and statistical analyses were performed using the SPSS program. If significant, Tukey's HSD post hoc analysis was performed. The P300 amplitude was determined as the highest peak between 200 and 500 ms, and latency was defined as the time lag between the onsets of stimuli to the P300-like peak amplitude.

## 3. Results

### 3.1. Behavioral Response

#### 3.1.1. Correct Lever Press Responses to Target versus Nontarget Auditory Tones

**Figure 2:**
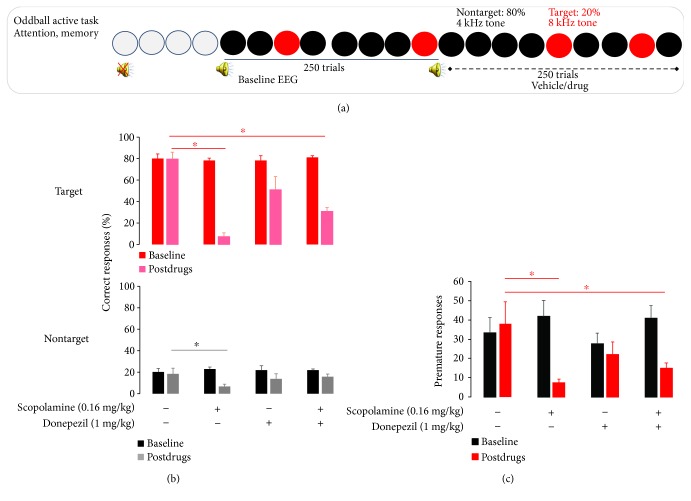
(a) Schematic presentation of the auditory active oddball protocol. The experiment started with a baseline run of 250 trials (20% oddball), followed by the administration of either vehicle or donepezil. Afterwards, scopolamine or vehicle was administered and the postdrug run continued with 250 trials (20% oddball). All recordings were carried out during the dark phase of the circadian rhythm. (b) Behavioral correct response during baseline and following pharmacological treatment in animals performing an auditory discrimination cognitive task to nontarget and target stimuli. (c) Behavioral premature response during baseline and following pharmacological treatment. Data are presented as mean ± SEM behavioral response baseline condition for each treatment group and to vehicle (*n* = 8), scopolamine (*n* = 5), donepezil (*n* = 7), and donepezil + scopolamine (*n* = 10). ∗ indicates significance difference from vehicle treatment.

Animals that followed all phases of the active oddball discriminant task (Figure 2(a)) showed high behavioral performance as animals markedly discriminated between target and nontarget tones by higher levels of correct pressing of the lever in target trials (≥80%) and lower level for incorrect lever presses (≤20%). Behavioral quantification of animals' ability to discriminate between target and nontarget tones is shown in Figures 2(b) and 2(c). In the vehicle condition, rats correctly responded with a lever press in 80% ± 6% for target trials (baseline 80% ± 4%), whereas correct lever presses were seen in 18% ± 5% for nontarget trials (baseline 20% ± 3%).

There was a treatment effect *F*(3, 32) = 20.8, *p* < 0.0001. When compared with vehicle, animals treated with scopolamine showed a significant decrease in behavioral performance as they correctly responded with a lever press in 7% ± 3% for target trials (baseline 78% ± 2%) and responses to the nontarget tones in 7% ± 2% (baseline 23% ± 3%). Donepezil reduced the correct responses to target tones with a lever press in 51% ± 12% of target trials (baseline 78% ± 4%), and although it reduced the responses to nontarget tone trials in 14% ± 5% (baseline 22% ± 1%), this was not statistically significant. In the combined pharmacological treatment group (Figure 2(b)), donepezil attenuated scopolamine-induced impairments in behavioral performance as animals treated with both drugs correctly responded with a lever press in 31% ± 3% for target trials (baseline 81% ± 2%) and responses to nontarget tones in 16% ± 3% (baseline 22% ± 1%) (*p* < 0.0001, resp.).

#### 3.1.2. Premature Lever Press Responses

Assessment of behavioral performance showed that the vehicle had no effects on premature lever press scores in 38.1 ± 11 (baseline: 33.4 ± 7.8) (Figure 2(c)). In pharmacologically challenged animals, there was a treatment effect *F*(3, 32) = 3.21, *p* = 0.03). When compared with vehicle, scopolamine significantly decreased premature lever press responses in 7.7 ± 1.5 (baseline: 41.9 ± 8.2) (*p* = 0.005). Donepezil had a slight effect on premature lever press responses 22.2 ± 6.3 (baseline: 27.9 ± 5.5), whereas a significant reduction in premature lever press responses was found in the combined pharmacological treatment in 15.2 ± 2.6 (baseline: 41.0 ± 6.4) (*p* = 0.02).

### 3.2. ERP Response

#### 3.2.1. P300-Like Amplitude and Latencies

**Figure 3:**
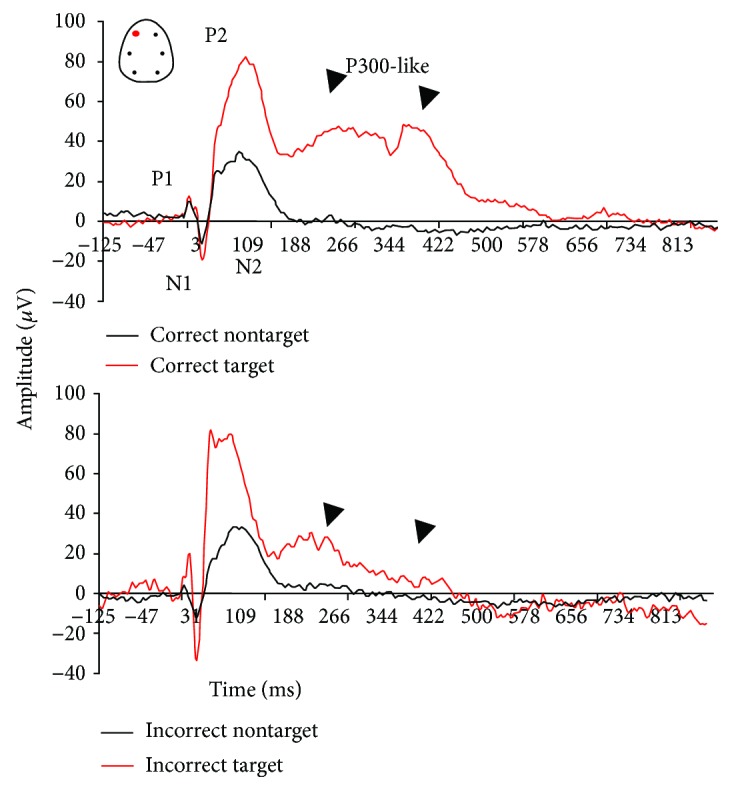
Grand average auditory-evoked waveforms recorded from the frontal left electrodes in rats performing in the auditory oddball discrimination task. Auditory potentials to target tone (red curve) stimuli consist of early positive (P1) and negative components (N1) and a late positive component (P300-like), which is preceded by a negative component (N2). In the late P300-like component, we could distinguish early and late positive waves occurring between 200 and 500 ms after target stimuli onset. Those components were smaller in AEPs to nontarget stimuli (black curve), and no P300 response was revealed by nontarget stimuli. This grand averaged waveform was calculated for all animals.

The active oddball AEP paradigm allowed the assessment of both preattention and attention-relevant cortical responses in rats performing a discriminant auditory task. All AEP responses presented here were derived from the frontal left electrode only. Under baseline conditions, rats that discriminate between stimulus tones showed complex components (P1, N1, P2, N2, and P300-like) in all AEP responses (Figure 3). The difference in the amplitude response to target versus nontarget tones is consistent with the ability to allocate attention resources required to discriminate and process target rare from nontarget stimulus tones. Animals that paid attention to the target stimuli generated two positive deflections between 200 and 500 ms after the onset of the tone stimuli, suggestive of early and late P300-like components (Figure 3).

**Figure 4:**
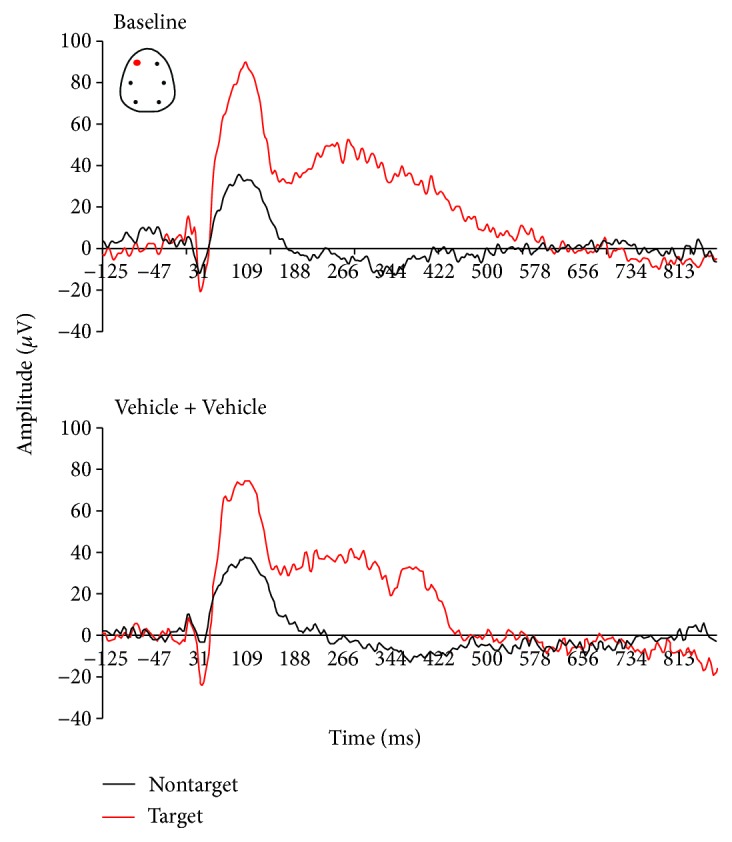
Grand average auditory-evoked waveforms recorded from the frontal left electrodes in rats performing an auditory discrimination task under baseline and following subcutaneous administration of vehicle (*n* = 8). Auditory potentials to target tones stimuli (red curves) showed similar evoked waveforms in both conditions.

**Figure 5:**
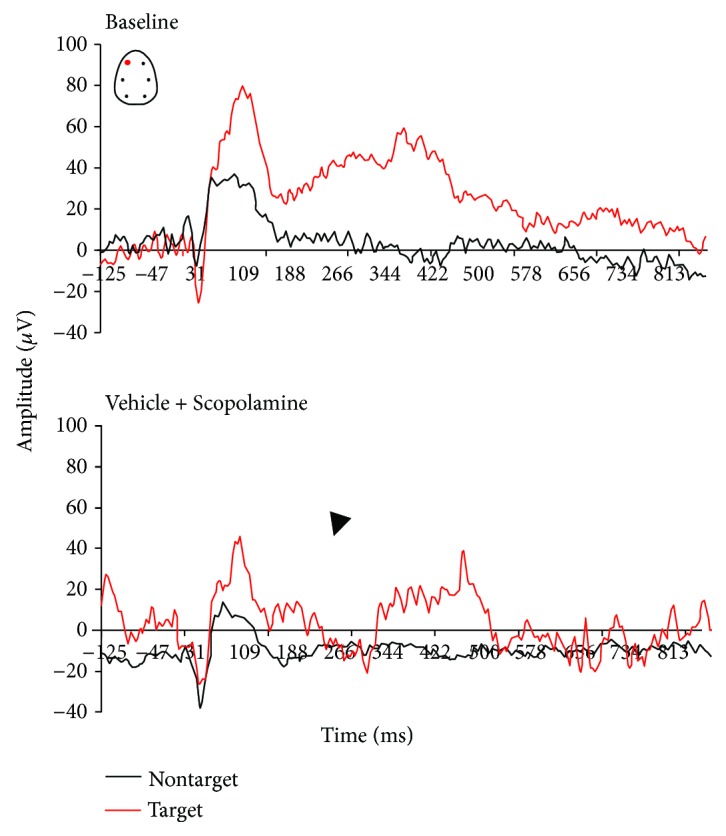
Grand average auditory-evoked waveforms recorded from the frontal left electrodes in rats performing an auditory discrimination task under baseline and following subcutaneous administration of scopolamine (0.16 mg/kg, *n* = 5).

**Figure 6:**
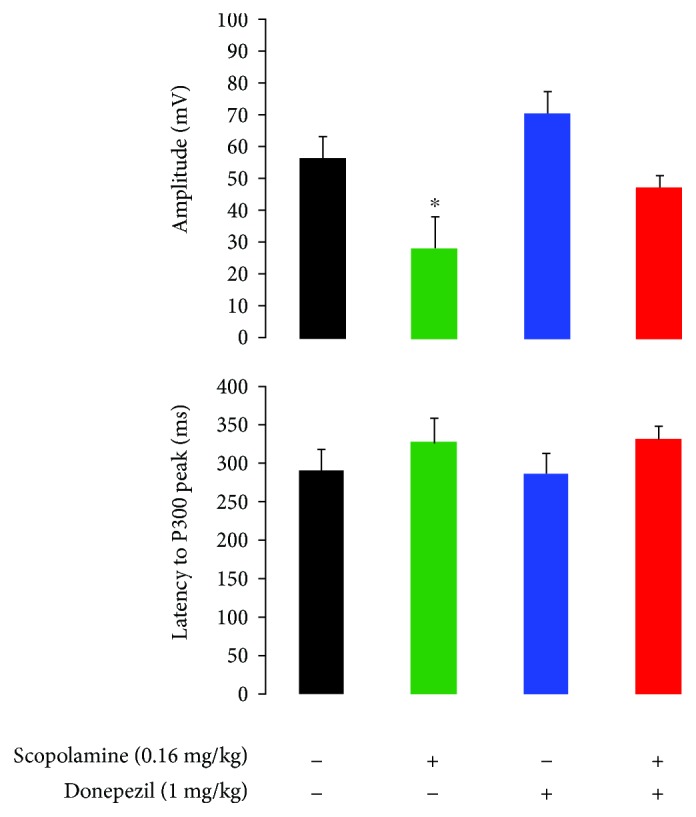
Difference in maximal peak amplitudes and latency to P300 response at 200–500 ms poststimuli onset in correct target trials following subcutaneous administration of vehicle (*n* = 8), scopolamine (0.16 mg/kg, *n* = 5), donepezil (1 mg/kg, *n* = 7), and combined donepezil and scopolamine (*n* = 10). Data is expressed as mean ± SEM. ∗ indicates significance difference vehicle treatment.

P300-like responses revealed significantly larger peak amplitudes in target trials compared to nontarget trials in baseline and vehicle conditions (Figures 3 and 4). There was a treatment effect at the amplitude of the P-300 like response (*F*(3, 25) = 3.91, *p* = 0.02). When compared to the vehicle condition, scopolamine consistently disrupted the AEP response to rare target stimuli and P300-like peak responses (*p* = 0.003) (Figures 5 and 6). No significant effect at the latency to apparent P300-like amplitude was observed (Figure 6).

**Figure 7:**
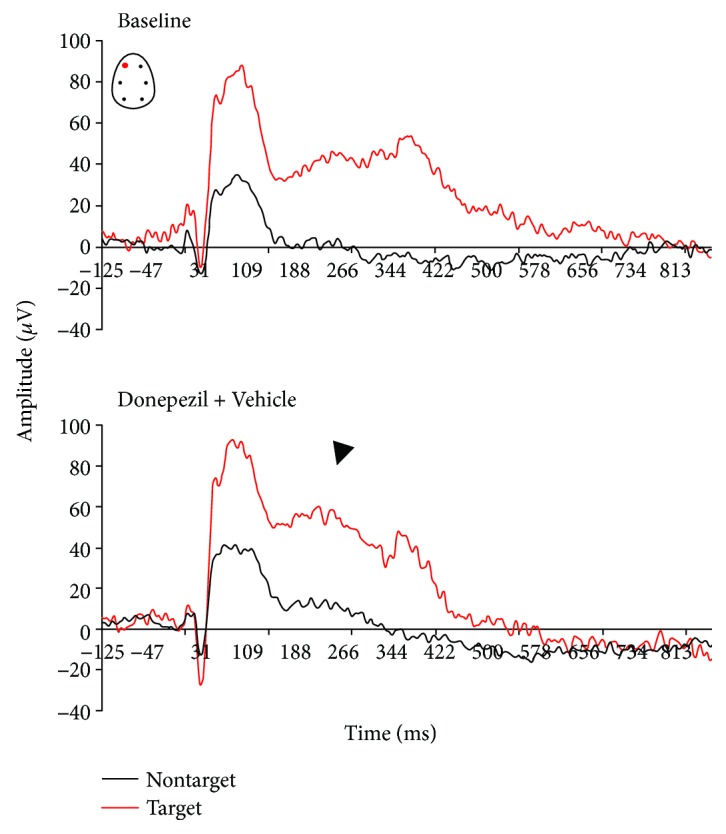
Grand average auditory-evoked waveforms recorded from the frontal left electrodes in rats performing an auditory discrimination task under baseline and following subcutaneous administration of donepezil (1 mg/kg, *n* = 7).

**Figure 8:**
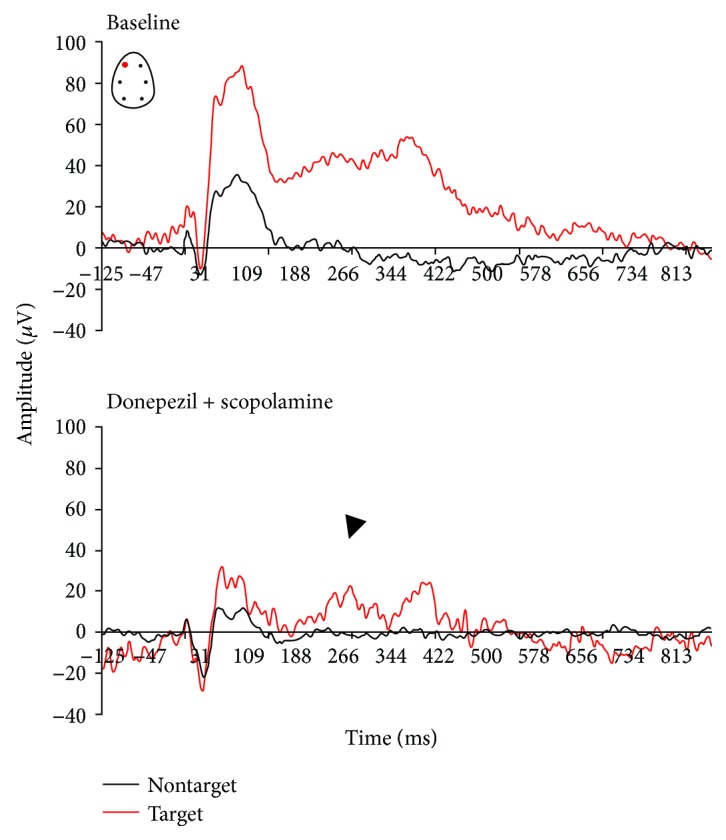
Grand average auditory-evoked waveforms recorded from the frontal left electrodes in rats performing an auditory discrimination task under baseline following subcutaneous combined pharmacological administration of scopolamine and donepezil (*n* = 10).

In donepezil-treated animals, a slight enhancement of the P300-like amplitude was observed (Figures 6 and 7). In the combined pharmacologically treated animals, donepezil slightly attenuated scopolamine-induced disruptive effects on complex components (*p* = 0.04) (Figures 6 and 8). No treatment effect on peak latency was observed in target trials (*F*(3, 25) = 1.6, *p* = 0.2).

### 3.3. EROS Response

**Figure 9:**
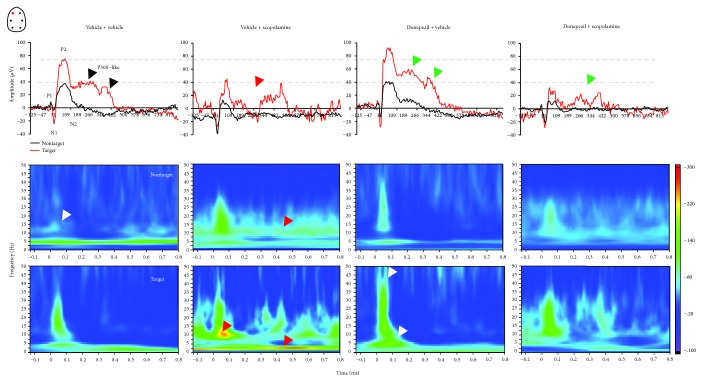
Relationship between ERP waveforms (top panels) and EROs (bottom panels) recorded from the frontal left electrodes in rats discriminating target and nontarget tones under pharmacological treatment: vehicle (*n* = 8), scopolamine (0.16 mg/kg, *n* = 5), donepezil (1 mg/kg, *n* = 7), and combined donepezil and scopolamine (*n* = 10).

The frontal P300-like response of the vehicle-treated animals performing the auditory discrimination cognitive task emerged with an event increase in cortical theta rhythm (Figure 9). The target stimuli did result in significantly more activation compared to nontarget stimuli. Scopolamine was associated with enhanced delta oscillatory rhythm (1–4 Hz) and alpha peak activity around 10 Hz to both target and nontarget stimuli. Donepezil elicited prominent increases in the slow theta (4–6 Hz) and beta-gamma oscillatory rhythms (12–40 Hz) observed at about 50 ms particularly for target stimuli. When combined, donepezil attenuated the effects of scopolamine on slow delta.

## 4. Discussion

In this active oddball study, rats could reach high performance levels after training to correctly respond by lever press after correct discrimination of target from nontarget tones. Task-relevant target tones evoked P300-like responses in animals that paid attention and reacted on the correct target stimuli, suggesting a modification of neuronal network activity due to enhanced attentional processes. Donepezil had no enhancing effects on behavioral performance, whereas the number of correct trials was lowered. However, scopolamine decreased correct lever pressing to target stimuli as well premature responses, which suggests that animals had forgotten what they had learnt. The decline in behavioral performance and correct detection of target stimuli was associated with reduced P300-like amplitude, which potentially reflects impaired synaptic strength.

The predictive validity of standard behavioral assays has been heavily criticized in contemporary cognitive neuroscience research because of poor translation of basic animal work into human outcomes and the lack of success in discovering a novel chemical entity targeting cognitive deficits [1–3]. LTP, which represents the basic mechanism underlying learning and memory, has widely been investigated at the cellular and molecular level in animals [10, 15, 55, 56]. Repetitive sensory stimulation of visual or auditory systems can induce noninvasively long-term potentiation-like effects in humans [17–19]. Cognitive ERP studies in neurodegenerative and psychiatric disorders revealed sensitivity and consistent abnormalities in several ERP components, each of which taps into different aspects of perceptual and/or cognitive processing. The temporal immediacy of the ERP response is particularly advantageous in the study of memory and attention as the memory encoding and retrieval process is very fast. The active oddball paradigms have been particularly important in clinical studies as they can provide a human model system allowing examination of synaptic plasticity of subjects with cognitive attentional deficits [19, 15–18, 20–59]. The P300 amplitude is thought to reflect attentional resource allocation, phasic attentional shifts, and working memory updating of the stimulus context, whereas its latency is thought to reflect processing speed or efficiency during stimulus evaluation [23]. Clinical studies have consistently shown great utility of this paradigm to study the brain's synaptic function and to probe subtle abnormalities of cognition; this was a motivation to demonstrate and differentiate these AEP components in the active oddball paradigm in rats and to evaluate the sensitivity to modulation of cholinergic neurotransmission.

Cholinergic transmission is thought to play major regulatory functions to memory encoding and consolidation [60–65], as well as to regulatory functions in motor behavior and reward-related behavior [66–70]. In animals and humans, the scopolamine cognitive challenge model has been widely used to examine the extent to which experimental compounds are hypothesized to act through modulation of cholinergic neurotransmission. Scopolamine is known to influence motor function, which might have interfered with learning response rules and number of correct response trials. In addition, experimental observations showed that visual information processing is modulated by cholinergic transmission [71, 72], and scopolamine has the potency to dilate the pupils, which may have impaired visual perception contributing to weak association between lever manipulation and task initiation/reward acquisition. However, scopolamine administered at higher doses (0.3–1 mg/kg) than that used in the present study neither impaired motor function in the open field paradigm nor impaired performance in visual discrimination tasks that used striped textures as stimuli [72]. Scopolamine has been shown to impair cognitive functions such as attention and working memory [73–75]. Systemic administration of scopolamine immediately after the learning trials impaired habituation of mice in the open field, and rats to odor discrimination as well as the retrieval of memory acquired from contextual fear conditioning [76–79]. In mice that were consecutively exposed to two learning situations, scopolamine selectively disrupts acquisition of new information, but had fewer effects on memory consolidation of previous memories [80]. In the present auditory discriminant task where animals were required to sustain their attention to detect a stimulus associated with food reward, reduction of the cholinergic tone impaired behavioral performance to first-learned situations and therefore it could be hypothesized that scopolamine disrupted attention to new information and working memory functions as the task execution in correct target trials was impaired.

The decrease in premature responses with scopolamine related to reduced number of executed trials and a tendency for a decrease in this parameter with donepezil may suggest an effect on impulse control. Impulsiveness is linked to faster response time and errors caused by premature response for incorrect trials to stimuli. Here, scopolamine decreased responses to target and nontarget stimuli as well as premature responses, which overall suggests that scopolamine had a stimulating effect. Accordingly, the wake-promoting agent modafinil that has been approved for treatment of excessive sleepiness facilitated the ability to inhibit impulsive response in rats performing in a 3-choice visual discrimination and sustained attention tasks, and in a healthy young man performing in delayed matching to sample, a decision-making and the spatial planning tasks task [81, 82]. In addition, donepezil reduced the magnitude of scopolamine-related behavioral performance impairments. The lack of full reversal of amnestic effects was probably related to drug temporal kinetics as scopolamine-induced peak cognitive impairments may last longer and the efficacy endpoints could be impacted by distinct pharmacokinetic profiles of donepezil and scopolamine.

The task-relevant target tone stimulus evoked complex potentials including peaks between 200 and 500 ms poststimuli suggesting a P300-like response.

The choice of the brain-recording sites was based on their principal roles in sensory processing, working memory, and attentional processes. The rat's frontal cortex, specifically the medial frontal cortex, is usually considered to be homologous to the dorsolateral prefrontal cortex of primates, which consists of the cortex homologous to the human anterior cingulate cortex (ACC) and dorsolateral prefrontal cortex [83, 84]. The cortex is hierarchically organized to serve higher-order integrative functions that are neither purely sensory nor purely motoric. The primary motor areas (M1/2), somatosensory cortex and parietal auditory association cortex, integrate sensory and auditory modalities and link auditory information to the planning of movement; they are thought to be the anatomical substrates of the highest brain function in humans' conscious thought, perception, and goal-directed action. In the present work, large evoked potentials were derived and were presented from frontal cortical areas.

In clinical ERP studies, two distinct P300 waveforms at different latencies are generally distinguished: P3a is elicited by unexpected stimuli with front central topography and thought to reflect stimulus-driven attentional capture or reorienting, whereas P3b reflects activation of attentional networks facilitating memory computations in temporal and parietal areas [23, 85]. In preclinical studies, both passive and active oddball paradigms were used to investigate ERP components including late positivity. In the passive oddball, where animals do not require extensive training, late positive potentials were identified in the mouse [40] and rat cortex [41–43]. In the active oddball paradigm, P300-like positive components with maximal voltage over the parietal cortex were generated by task-relevant stimuli in rats [41–87], and P300-like responses were sensitive to pharmacological modulation of the cholinergic tone [46].

Here, novelty-related amplification of the P300-like response was larger in the frontal cortex of rats that correctly detected task-relevant stimuli. Our results further extend previous preclinical reports by showing that late AEP-positive components can be generated in the rat cortex in response to rare target versus frequent nontarget stimuli [41, 45, 50]. Two components have been described in separate oddball ERP P300 studies in rats [41, 43, 45, 50, 88, 89]. The early P3-positive component has been described around 240 ms [88], whereas the late P3 component was found at 380 ms [50]. A subsequent study described both early P3E and late P3L in response to correct deviance detection in frontal and parietal cortical areas in rats [87]. The deviance detection presented here was dominated by two positive peaks at different latencies, that is, an early component at ∼250–270 ms and a late component at >300 ms after oddball stimuli, which corroborate the existence of the two P300-like distinct peaks in rats [87].

Clinical observations support an important role of cholinergic transmission in the generation of P300 response in healthy men [48, 90, 91], and those parameters are impaired in AD patients generally related to a loss of cholinergic neurotransmission [49, 59]. Endogenous late ERP positivity is influenced by cholinergic transmission in primates [92], in rats, and in cats [38, 93]. The present results show that P300 late components were very sensitive to modulation of cholinergic neurotransmission. Scopolamine severely disrupted AEP complex response to correct target and nontarget tones suggesting some impairments in working memory traces of frequent and nonfrequent tones. The effects we observed indicate a frontal contribution of muscarinic receptors to the early P300-like component, and the limited number of correct responses to target stimuli due to alteration in cholinergic tone is likely associated with reduced allocation of attentional resources required to evaluate and discriminate the target tone.

Donepezil did not fully reverse the amnestic action of scopolamine in a variety of experimental cognitive tasks in animals and humans [93, 94]. In AD patients, donepezil decreased P300 latency, which was associated with improvement in remote memory, recent memory, and orientation [49, 59, 38]. In the rat model of basal forebrain cholinergic degeneration, donepezil attenuated deficits in P300-like response [46]. In the present study, donepezil increased the maximal amplitude of P300-like components and partially attenuated scopolamine-induced reduction of the P300-like amplitude. No significant effect on P300-like latencies indicates that the P300-like amplitude is a better marker of cholinergic treatment-dependent maintenance of active attention.

Accumulating evidence supports the relevance of network oscillation synchrony in normal brain function and during cognitive processes [95, 96]. Target tone responses were characterized by increased phase-locking of spectral power in slow theta and beta rhythms for target stimuli compared to nontarget stimuli. Donepezil evoked slow theta oscillations associated with increases in beta and gamma-band rhythm, with the magnitude being pronounced for target versus nontarget stimuli. In contrast, scopolamine abolished this slow theta rhythm and shifted the event-evoked oscillation towards the alpha rhythm. Pharmacological studies have revealed two subgroups of theta network oscillations: atropine-sensitive type 2 and atropine-resistant 1. The theta rhythm type 2 (<6 Hz) is also abolished by the muscarinic antagonist scopolamine and directly related to sensory stimulation but not motor activity, whereas the atropine-resistant type 1 is directly related to motor behavior [97]. Therefore, the present study provides evidence that the evoked slow theta rhythm by donepezil, which is inhibited by scopolamine, is associated with initial processing phases of target auditory tone information. Accordingly, the P300-like peak response to target stimuli was not at the theta frequency at 200–500 ms after stimulation. This agrees with a previous study in a visual three-stimulus oddball task, in which theta activity is more related to novel rather than target stimuli [46]. Future experiments will evaluate network frequency oscillations by an auditory stimulus novelty embedded to target/nontarget tones at 200–500 ms poststimuli onset in the auditory three-tone oddball.

Electroencephalography has revealed abnormalities in ERPs evoked by auditory stimuli in humans with neurodegenerative and neuropsychiatric disorders, indicating disruption of neural connectivity. Similar abnormalities in auditory-evoked ERPs have been observed in animal models of these brain disorders, suggesting that ERPs have the potential to provide a translational biomarker for those brain disorders. One of the widely used cognitive assays to assess drug effects in normal animals is to manipulate neural transmission to mimic abnormalities that are observed in the disease states. These disease models attempt to capture at least some part of the disease process that manifests itself in cognitive deficits. A common approach in modeling AD has been to disrupt the function of the cholinergic system, which is based on the observations that the most consistent neurochemical change in AD is a reduction of cholinergic markers in the forebrain and that muscarinic and nicotine cholinergic antagonists impair performance in cognitive tasks in both experimental animals and healthy men. The present findings further confirm the contribution of cholinergic neurotransmission in the generation of the P300-like response in the discriminant performance task. This active oddball paradigm in rats, in which the correct deviance detection was dominated by P300-like components, may have translational value to assess biological circuits and/or novel pharmacological agents interacting with the cholinergic transmission to facilitate and/or suppress synaptic responses associated with increased allocation of attentional resources.

## Data Availability

All data are fully available without restriction.
